# A Robust Biomimetic Superhydrophobic Coating with Superior Mechanical Durability and Chemical Stability for Inner Pipeline Protection

**DOI:** 10.1002/advs.202305839

**Published:** 2024-01-15

**Authors:** Xuerui Zang, Jiang Bian, Yimeng Ni, Weiwei Zheng, Tianxue Zhu, Zhong Chen, Xuewen Cao, Jianying Huang, Yuekun Lai, Zhiqun Lin

**Affiliations:** ^1^ College of Pipeline and Civil Engineering China University of Petroleum (East China) No. 66, West Changjiang Road, Huangdao District Qingdao 266580 P. R. China; ^2^ College of Chemical Engineering Fuzhou University Fuzhou 350116 P. R. China; ^3^ College of Chemical and Biomolecular Engineering National University of Singapore Engineering Drive 4 Singapore 117585 Singapore; ^4^ Qingyuan Innovation Laboratory Quanzhou 362801 P. R. China; ^5^ School of Materials Science and Engineering Nanyang Technological University 50 Nanyang Avenue Singapore 639798 Singapore

**Keywords:** bionic microstructure, mechanical durability and chemical stability, porous structure, superhydrophobicity, wear resistance

## Abstract

Durable superhydrophobic anti‐erosion/anticorrosion coatings are highly demanded across various applications. However, achieving coatings with exceptional superhydrophobicity, mechanical strength, and corrosion resistance remains a grand challenge. Herein, a robust microstructure coating, inspired by the cylindrical structures situated on the surface of conch shell, for mitigating erosion and corrosion damages in gas transportation pipelines is reported. Specifically, citric acid monohydrate as a pore‐forming agent is leveraged to create a porous structure between layers, effectively buffering the impact on the surface. As a result, the coating demonstrates remarkable wear resistance and water repellency. Importantly, even after abrasion by sandpaper and an erosion loop test, the resulting superhydrophobic surfaces retain the water repellency. The design strategy offers a promising route to manufacturing multifunctional materials with desired features and structural complexities, thereby enabling effective self‐cleaning and antifouling abilities in harsh operating environments for an array of applications, including self‐cleaning windows, antifouling coatings for medical devices, and anti‐erosion/anticorrosion protection, among other areas.

## Graphical abstract

Inspired by the cylindrical structures situated on the conch shell, a flexible and sturdy anti‐erosion/anticorrosion superhydrophobic coating is synthesized. The coating exhibited a remarkable 71％ reduction in erosion rate, measuring at 27.22 nm/s in contrast to the 316L steel substrate. Additionally, the coating increased the corrosion potential from −0.08 V to −0.02 V, underscoring its impressive performance in combating erosion/corrosion.

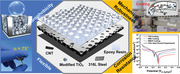

## 1 Introduction

Protective coatings for gas and liquid transportation pipelines (316L metal) play a vital role in safeguarding against damage caused by particle collision and corrosion from the conveyed medium.^[^ ^1^, ^2^, ^3^ ^]^ Recently, there has been a notable focus on hydrophobic coatings due to their potential to markedly enhance corrosion resistance by minimizing contact between the medium and the material surface.^[^ ^4^, ^5^, ^6^, ^7^ ^]^ Nonetheless, the limited applications of hydrophobic coatings in the pipeline industry stems from their inherent issues of fragile surface texture and inadequate wear resistance.^[^ ^8^, ^9^ ^]^ In light of the Cassie‐Baxter model,^[^ ^9^, ^10^ ^]^ which suggests that reducing the solid–liquid contact area can lead to an increase in the apparent contact angle and a decrease in the rolling angle:

(1) $$\;\cos\theta_{r}=f\left(1+\cos\theta_{y}\right)-1$$

where *θ*_r_ represents the apparent contact angle, *θ*_y_ denotes the Young's contact angle, *f* refers to the liquid‐solid contact friction, and *f* < 1. Notably, the intrinsic contact angle *θ*_y_ between the solid and the droplet is a constant value. Consequently, as the apparent contact angle increases, the value of *f* decreases, signifying a smaller actual contact area between the droplet and the solid surface.

As such, as stated by $p=\frac{F}{S}$, where *p*, *F*, and *S* represent the pressure on the contact surface, external load, and contact area, respectively, it is clear that microstructures experience higher local pressures due to reduced contact area, rendering them more susceptible to wear and other forms of damage.^[^ ^11^ ^]^ Consequently, a distinct contradiction arises between the requirements for superhydrophobicity and mechanical robustness. To date, several strategies have been explored to address this issue. For instance, wear resistance can be enhanced through the incorporation of mixed micro/nano fillers.^[^ ^12^ ^]^ Furthermore, various filler shapes, including fibrous, granular, and square, can be introduced to improve shock wave absorption capability.^[^ ^13^ ^]^ Binders are utilized as the continuous phase to reinforce the binding between nanoparticles (NPs) and substrate.^[^ ^14^, ^15^ ^]^ However, the ability to attain the coatings possessing the concurrent superhydrophobicity, mechanical robustness,^[^ ^16^ ^]^ wear resistance,^[^ ^17^ ^]^ chemical resistance,^[^ ^18^ ^]^ and substrate adhesion^[^ ^19^ ^]^ remains a substantial challenge. In this context, a biomimetic microstructure strategy may stand out as a promising route to coatings with enhanced mechanical and chemical properties noted above.

Herein, we developed an unconventional biomimetic microstructure coating with a porous design (denoted BMCP) to mitigate erosion and corrosion damage in gas transportation pipelines via judiciously integrating three concerted strategies involving microscale metallic surface structuring, porous buffer layer introduction, and steel fiber/rebar‐reinforced concrete structure‐resembled construction. In microscale metallic surface structuring, biomimetic microstructures are created using metal 3D printing to enhance the wear resistance of the coating surface. Various biological surface microstructures known for their excellent wear resistance, including cylinders (conches), triangular prisms (pangolins), cuboids (crocodiles), and quadrangular prisms (pythons), are designed and 3D‐printed facilely, forming a bionic layer. In order to incorporate a porous buffer layer, a poly(dimethyl siloxane) (PDMS) sponge with a porous structure is produced using citric acid monohydrate (CAM) as a pore‐forming agent. The attained buffer layer, designed to improve mechanical resistance, imparts the coatings with outstanding impact resistance.^[^ ^20^ ^]^ To enable the steel fiber/rebar‐reinforced concrete‐resembled structure,^[^ ^21^ ^]^ a strong superhydrophobic coating by exploiting carbon nanotubes (CNTs), lauric acid (LA)‐modified TiO_2_ NPs, and epoxy resin (EP) is formulated. The selection of EP is due to its mechanical and chemical robustness as well as strong substrate adhesion. The high aspect ratio and flexibility of CNTs facilitate the bonding between dissimilar materials, while the modified TiO_2_ NPs provide texture control and low surface energy to enhance surface hydrophobicity. The resulting BMCP coating manifests not only exceptional mechanical durability, including impact resistance, wear resistance, and hydrophobic stability, but also superior bendability, chemical robustness, corrosion resistance, and strong bonding, highlighting its great potential for a set of applications that require long‐term service in harsh environments, such as ship maintenance, antifouling coatings for optical sensors, self‐cleaning windows, and biomedical devices.

## 2 Results and Discussion

### 2.1 Robust Route to Biomimetic Superhydrophobic Coating

**Figure 1:**
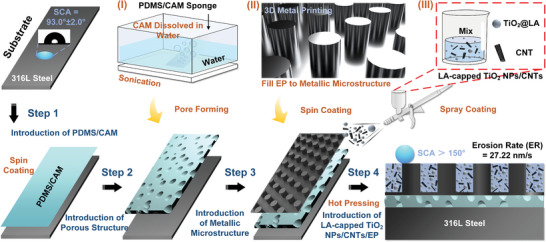
Biomimetic microstructure coating with a porous design (BMCP). Stepwise representation of the route to BMCP coating (lower right panel). Step 1: PDMS/CAM was spin‐coated on the original 316L steel substrate. Step 2: Pore buffer layer was generated by ultrasound in water. Step 3: The opposite side of the buffer layer was bonded to the substrate and metal microstructure through pure PDMS. Step 4: EP and LA‐capped TiO_2_ NPs/CNTs were filled into the gaps of microstructure through spin‐coating and spraying, respectively, followed by hot‐pressing to generate final product. Illustration I): After contact with water, CAM is decomposed, and pores are formed in PDMS. Illustration II): Filling uncured EP into gaps in metal microstructures through spin‐coating. Illustration III): After illustration II, CNTs are evenly embedded in the uncured EP through spraying.

**Figure** **1** depicts the coating process. First, a mixture of high‐temperature‐cured PDMS and CAM (at a ratio of PDMS: CAM = 1:1, Table S1, Supporting Information) was subjected to ultrasonication in an aqueous solution to create pores, forming a PDMS sponge (see Experimental Section in Supporting Information). Then, PDMS was spin‐coated onto both the 316L stainless steel substrate and the bionic layer which was 3D‐printed using 316L powder. The optimized bionic layer and 316L substrate were then respectively bonded to the opposite sides of the PDMS buffer layer. Subsequently, EP was spun onto the bionic layer to impart a complete filling of the gaps between the metallic microstructures. The TiO_2_ and CNTs at a mass ratio of 2:1 (Table S2, Supporting Information) were dispersed in water with vigorous stirring and sprayed onto the unsolidified EP. Finally, the superhydrophobic nanocomposites coating was yielded through hot pressing at 25 kPa and 120 °C.

The successful chemical grafting of LA was confirmed by FTIR measurement. Figure S1A (Supporting Information) displays the IR spectra of LA, pristine TiO_2_ NPs, and LA‐modified TiO_2_ NPs. In addition to the Ti─O bond at 628 cm^−1^, characteristic bands corresponding to O─H groups at 1628 and 3591 cm^−1^, as well as C─H groups at 2827 and 2939 cm^−1^, are evident. A peak at 1725 cm^−1^ appeared on the LA‐modified TiO_2_ corresponds to the ‐COOH stretching vibration, indicating the success in tethering the surface of TiO_2_ NP with LA. Figure S1B (Supporting Information) shows the energy dispersive X‐ray spectroscopy (EDS) analysis on a cylinder‐shaped microstructure, revealing the presence of C, Cr, Ni, Mn, and Mo, which originate from the 316L powder used for 3D printing of microstructures.

### 2.2 Rational Design and Crafting of BMCP with Concurrent Outstanding Wear Resistance and Superhydrophobicity

**Figure 2:**
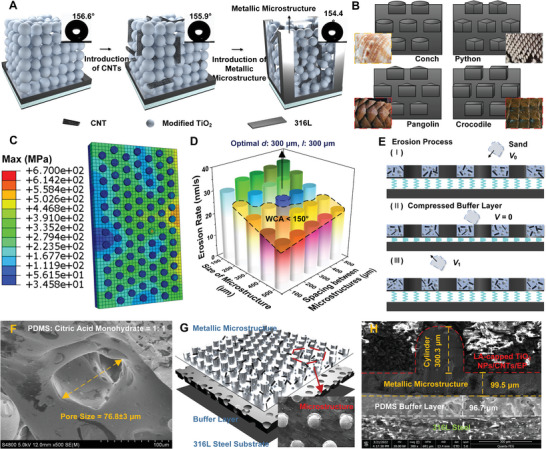
Rational design and crafting of biomimetic microstructure coating with a porous design (BMCP) to yield optimal performance (i.e., simultaneous outstanding erosion property and superhydrophobicity). A) Schematic of a unit volume of the BMCP coating containing cylinder‐shaped microstructure and LA‐capped TiO2 NPs/CNTs situated on a PDMS buffer layer/316L steel substrate. B) Introduction of various biomimetic microstructures, namely, hexagonally arranged cylinders (resembling the conch shell), quadrangular prisms (resembling the python skin), triangular prisms (resembling the pangolin skin), and cuboids (resembling the crocodile skin). C) Schematic diagram of a BMCP coating used in simulation. D) The erosion rate of BMCP coatings with cylinder‐shaped metallic microstructures of different cylinder size and spacing between adjacent cylinders, where an optimal microstructure with concurrent lowest erosion rate and superhydrophobicity is identified (labeled in blue with a d of 300 µm and a l of 300 µm). E) Schematic illustration of the introduction of PDMS buffer layer that offers the improved erosion resistance upon the impact from sands inside pipeline. F) SEM image of porous PDMS buffer layer. The average pore size is 76.8 ± 3 µm. G) Overall structure of the BMCP coating (inset shows a scanning electron microscope (SEM) image of cylinder‐shaped metallic microstructure). H) Cross‐sectional SEM image of a BMCP coating, showing dimension and thickness of constituents.

The significant enhancement in wear resistance of the BMCP coating can be attributed to the judicious crafting of microscopic structures (**Figure** **2**A). These regularly distributed microstructures resemble the “retaining walls” (right panel in Figure 2A), which prevent “soil” (i.e., LA‐capped TiO_2_ NPs/CNTs) displacement due to confinement and thus maintain its stability under external force (i.e., impact). Consequently, such periodically patterned microstructures effectively mitigate the removal of LA‐capped TiO_2_ NPs/CNTs from the coating surface caused by the prolonged erosion.

Inspired by biological microstructure surfaces with diverse shapes and distributions, four types of biological skin with excellent wear resistance were simplified as cylinders (resembling the conch shell), triangular prisms (resembling the pangolin skin), cuboids (resembling the crocodile skin), and quadrangular prisms (resembling the python skin) (Figure 2B). The size and shape of the surface microstructures were optimized using ABAQUS finite element simulation (Figure 2C; Figures S2–S4, Supporting Information). In contrast to common microstructure coatings such as armors arranged in a square manner (i.e., squared arrangement)^[^ ^22^ ^]^ (Figure S4, Supporting Information), the dislocation arrangement (i.e., hexagonally arranged; Figure S3, Supporting Information) offers a distinct advantage in preventing particles from displacement and damage during impact (Figure S5A, Supporting Information), where smaller structural surface force is resulted in (open circles in Figure S5A). It is also notable that among all hexagonally arranged microstructures, the cylinder‐shaped microstructure experiences the smallest structural surface force, reflecting the optimal microstructure to sustain the wear (i.e., the best wear resistance). Thus, the cylinder‐shaped microstructures (i.e., conch‐shell‐inspired microstructures) were implemented in all studies discussed below.

The elastic response of the coating, influenced by the surface microstructure layer, was also evaluated. Figure S5B (Supporting Information) depicts that, among a set of hexagonally arranged microstructures, the microstructures with a diameter *d* of 100 µm and a spacing *l* between adjacent microstructures of 500 µm (i.e., marked with a black dashed open circle) experience an optimal structural surface force. It is important to note that in order to render excellent wear resistance and superhydrophobicity (with a water contact angle, WCA > 150°) concurrently, the microstructure with a *d* of 300 µm and a *l* of 300 µm was identifies by experiment via study of the erosion rate of the coating, manifesting a highest resistance to the tested load while superhydrophobic (Figure 2D).

In our work, to further decrease the external force on particles, a porous PDMS buffer layer integrated with various microstructures discussed above was deliberately introduced to enhance the erosion resistance (Figure 1, step 2 and Figure 2E). Upon impact, the PDMS sponge layer absorbs a portion of the external load via deformation, resembling a spring absorbing energy upon compression. The pore size in the buffer layer is illustrated in Figure 2F. Notably, in comparison to pure PDMS, the addition of CAM to create micropores improved the impact resistance of the coating (Table S3, Supporting Information). The optimal buffer layer thickness and the mixing ratio of PDMS and CAM are shown in Figure S6 (Supporting Information).

Figure 2G illustrates the overall coating structure, comprising two layers with a top layer consisting of metal microstructures of varied shapes and LA‐capped TiO_2_ NPs/CNTs and a bottom layer of PDMS sponge that connects with the 316L steel substrate. Cross‐sectional image and surface topology of the coating structure are shown in Figure 2H and Figures S7 and S8 (Supporting Information), respectively. The size of the cylinder‐shaped microstructure is ≈300.3 µm, and the thickness of the PDMS buffer layer is ≈96.7 µm. It is worth noting that the binding of the PDMS buffer layer with the 316L metal substrate and the microstructure/LA‐capped TiO_2_ NPs/CNTs is outstanding, with no discernible cracks or delamination.

### 2.3 Mechanical Durability of BMCP

**Figure 3:**
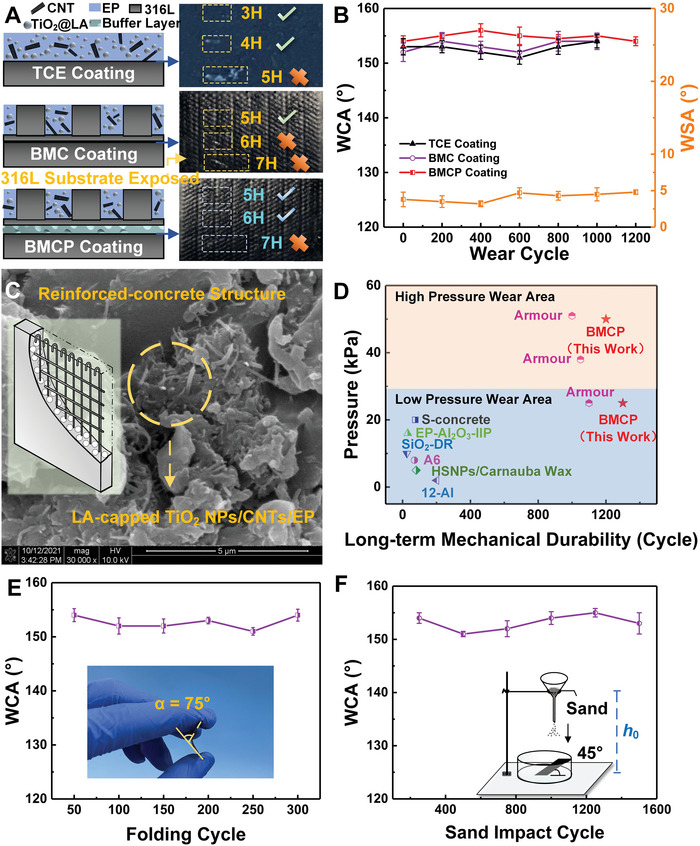
Investigation into the mechanical durability of various coatings. A) The influence of the pencil hardness test on various coating surface. The check and cross marks represent the coating surface remained intact and scratched, respectively. B) Relationship between the wear cycle and the hydrophobic property of various coatings. C) The mixing of CNTs and LA‐modified TiO_2_ is similar to the reinforced concrete structure, resisting the impact of sand particles on the surface of BMCP coating. D) Comparison of abrasion tests with references (i.e., Armor,^[^ ^22^ ^]^ HSNPs/carnauba wax,^[^ ^29^ ^]^ S‐concrete,^[^ ^30^ ^]^ 12‐Al,^[^ ^31^ ^]^ A6,^[^ ^32^ ^]^ SiO_2_‐DR,^[^ ^33^ ^]^ EP‐Al_2_O_3_‐IIP^[^ ^34^ ^]^).^[^ ^22^, ^29^, ^30^, ^31^, ^32^, ^33^, ^34^ ^]^ E) The influence of bending test at 75° bending angle (inset) on the wettability of coating. F) The wettability of coating as a function of the sand impact cycle. Inset depicts the sand impact test.

The scratch resistance of the BMCP coatings was conducted using the pencil hardness test (**Figure** **3**A) with the hardness from the hardest (9H) to the softest (9B). Comparing the coating to the 316L steel substrate (a hardness of 4H), three modified coatings were assessed, namely, the BMCP coating, the BMCP coating without a porous PDMS layer (denoted BMC coating), and the TiO_2_@LA/CNTs/EP coating (i.e., without the 316L steel substrate and PDMS buffer layer; denoted TCE coating). The BMCP coating achieved a pencil hardness of 6H, whereas the TCE and BMC coatings only reached 4H and 5H, respectively. This demonstrates the effectiveness of the biomimetic conch‐shell‐inspired microstructure and PDMS sponge in boosting the mechanical properties.

Wear resistance is a crucial indicator for evaluating the quality of coatings, particularly for superhydrophobic surface. In our study, the superhydrophobic coating underwent abrasion using 600‐grit sandpaper, with each cycle involving a 20 cm movement under a load of 2000 g (or 49 kPa). The resistance of the bionic coating was evaluated after every 100 abrasion cycles based on the wetting properties measured. As depicted in Figure 3B, the WCA and water sliding angle (WSA) of the BMCP coating after 1200 wear cycles remained at 153° and 4.8°, respectively. In contrast to the BMC and TCE coatings, the BMCP coating exhibited an extended lifespan of 200 additional cycles, highlighting its remarkable wear resistance. The laser scanning confocal microscopy study confirmed the stability of the BMCP coating as a direct consequence of the synergy of the armor characteristic of metallic microstructures, the buffering effect of porous PDMS layer, and the reinforcing effect of the LA‐capped TiO_2_ NPs/CNTs resembling the steel fiber/rebar‐reinforced concrete structure (Figure 3C). The performance of our BMCP coatings in terms of abrasion resistance was benchmarked over the reported results (Figure 3D), including the coatings with armor structures known for significant wear resistance.^[^ ^22^ ^]^ Clearly, our BMCP coatings manifested the outstanding resistance, outperforming the reported coatings in long‐term mechanical durability (Figure 3D).

The enhancement of coating robustness stems from various contributing factors. Primarily, the phenomena like friction, collision, impact, or compression can be dissected into two primary forms of surface damage, that is, shear load aligned parallel to the surface and compression load perpendicular to it.^[^ ^23^, ^24^ ^]^ In addressing shear loads, biomimetic conch surfaces featuring armor‐like structures unequivocally assume a pivotal role. Functioning as receptacles for carrying micro and nano particles, this micro cylindrical array profoundly aids in resisting the displacement (dislodge) of surface NPs when subjected to shear loads, consequently substantially mitigating the extent of damage to surface nanostructures.^[^ ^25^ ^]^ Additionally, the fibrous structures of CNTs serve to deter NPs aggregation and significantly reinforce the bonding strength between EP and NPs.^[^ ^26^ ^]^ Under shear loads, these structures undergo a process where fibers are pulled out and stretched, necessitating a notable increase in energy consumption. This characteristic proves advantageous in impeding the development and spread of surface microcracks, thereby bolstering mechanical properties of the coatings, such as fracture toughness.^[^ ^27^ ^]^ Lastly, sponge‐like structures characterized by a low elastic modulus exert an obvious reduction in material surface damage due to compressive loads, thereby converting instantaneous impacts into prolonged oscillation processes.^[^ ^28^ ^]^ According to the momentum theorem, given a constant external load, a longer impact process undergone by the material surface corresponds to a lesser force it endures.

Furthermore, as shown in Figure 3E, the BMCP coating displayed excellent flexibility and fatigue resistance, as it could be repeatedly folded from 0° to 75° for 300 cycles without delamination or cracking. The superhydrophobicity of the coating was well retained throughout the folding process, signifying its suitability for curved substrates such as the inner walls of pipes. Additionally, the BMCP coating showed negligible changes in the CA after 1500 cycles of the sand impact test from a height of 1 m (Figure 3F).

### 2.4 Wettability and Anti‐Corrosion Characteristic of BMCP

**Figure 4:**
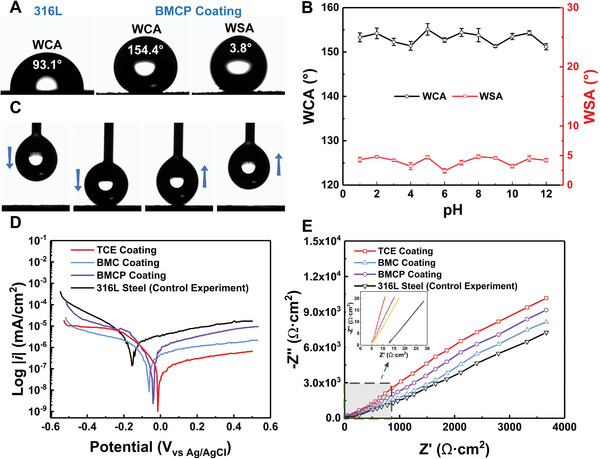
Characterization of superhydrophobicity and corrosion resistance. A) Water contact angle of 316L steel and the BMCP coating surfaces, respectively. B) WCA and WSA of the BMCP coating at different pH values (adjusted by adding HCl or NaOH). C) Digital images of water droplet, demonstrating the low adhesion of the BMCP coating to water. The corrosion resistance of various coating, revealed by D) polarization curves and E) impedance spectra.

The as‐prepared BMCP coating demonstrated an outstanding superhydrophobicity, achieving a WCA of up to 154.4° and a WSA as low as 3.8°. In contrast, water droplets on an uncoated 316L steel surface exhibited a strong adhesion, with a WCA of 93.1° (**Figure** **4**A). The relationship between the coating wettability and tape peeling is illustrated in Figure S9A (Supporting Information). The WCA of the TCE coating with the buffer layer decreased to 151° after 40 peeling cycles and further to 138.7° after 50 peeling cycles. In contrast, the BMCP coating retained a WCA of 152.7° after 50 cycles of peeling, and the peeling process did not result in the loss of LA‐modified TiO_2_ NPs/CNTs in the coating, suggesting a strong adhesion among the LA‐modified TiO_2_ NPs/CNTs, EP and conch‐shell‐inspired microstructure (Figure S9B, Supporting Information). Furthermore, the WCA and WSA of the coating showed minimal variation with pH change, indicating its superhydrophobic behavior toward liquids with different acidity and alkalinity (Figure 4B; Figures S10 and S11, Supporting Information). The adhesion of water droplets to the coated surface was extremely low, as demonstrated by the ability of a droplet collected by a syringe needle to freely leave the coating without residue (Figure 4C).

To analyze the resistance of the coating to electrochemical corrosion, an electrochemical study was conducted in 3.5 wt.% NaCl electrolyte. Compared to the planar 316L steel substrate, the corrosion potential of the three modified coatings (i.e., TCE, BMC, and BMCP; Figure 3A) increased from −0.08 to −0.02 V. Additionally, the corrosion current of the BMCP coating was decreased by 7.025 × 10^−7^ A cm^−2^, signifying a lower tendency for corrosion reactions and a reduced corrosion rate (Figure 4D). It is noteworthy that the ability of the BMCP and BMC coatings to inhibit corrosion is slightly lower than that of the TCE coating, representing a 0.04 V decrease in the self‐corrosion potential. Such a decrease in corrosion potential can be attributed to the direct contact between a small amount of bare metallic microstructure and the corrosion solution. As illustrated in Figure 4E, the diameter of the capacitance rings in the Nyquist plot progressively increased from bare 316L steel to BMC, BMCP, and TCE coatings. In general, a larger capacitance ring signifies a greater corrosion resistance, reflecting the polarization resistance of the working electrode.^[^ ^35^ ^]^

### 2.5 Erosion Resistance of BMCP

The erosion test of solid particles (i.e., quartz sand) on a pipeline elbow was conducted using an erosion loop device (Figure S12A, Supporting Information). The loop setup included a water tank, mixer, flow acquisition system, flow velocity, pressure meters, visual erosion loop, test elbow, compressor with buffer tank, and sections for gas–liquid mixing and separation. The exposed area of the test samples was 8 mm × 6 mm, with a thickness of 0.6 mm. Quartz sand with a particle size of 280–320 µm and a density of 2650 kg m^−3^ was employed in the experiment (Figure S12B, Supporting Information). The sand was transported into the testing loop by the compressor, with an inlet velocity of 20 m s^−1^ and a mass flow rate of sand particles set at 0.2 kg s^−1^ (Figures S12C,D, Supporting Information). The erosion test was conducted for a duration of 24 h.

The weight change of the sample before and after erosion was measured, and the erosion rate (ER) was calculated using the equation:^[^ ^36^, ^37^, ^38^ ^]^

(2) $$ER=\frac{1}{3600000}\times \frac{w_{0}-w_{1}}{S\cdot t\cdot \rho_{w}}$$

where *w*_0_ represents the initial mass of the sample, *w* is the mass of the sample after the test, *S* is the working area of the sample, *t* is the erosion time, and *ρ*_w_ is the density of the target material.

The average density of the coating can be determined by the following equation:

(3) $$\rho_{w}=\rho_{\mathrm{water}}\frac{m_{2}-m_{3}}{m_{1}-m_{3}}$$

where *m*_1_ is the quality of the beaker filled with water, *m*_2_ is the quality of the coating after placing it in a beaker filled with water, *m*_3_ is the total mass of the beaker and the remaining water after removing the coating, and *ρ*_water_ is the density of water.

**Figure 5:**
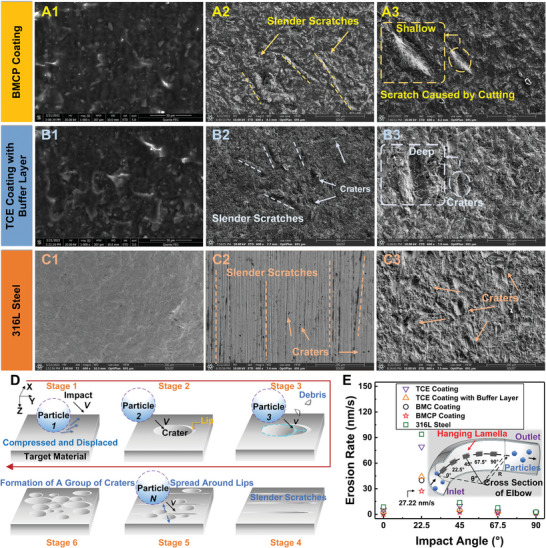
Erosion resistance of various coatings. A1–C3) SEM images of the BMCP coating, the TCE coating with a buffer layer, the 316L steel sample before (A1, B1, and C1) and after (A2‐A3, B2‐B3 and C2‐C3) erosion. D) Stepwise representation illustrating the erosion mechanism on coating surface. E) Effect of various coating structures on the erosion rate. Inset shows the section of the elbow with the trajectory of particles inside.

**Figures** **5**A1–C3 illustrates the relationship between the erosion duration and the surface morphology of three different materials, namely, the BMC, BMCP, and TCE coatings on the 316L steel substrate. After a 12 h erosion cycle, noticeable slender scratches were appeared on the surface of the BMCP coating, which were subsequently covered by disordered craters after 24 h. This formation mechanism can be rationalized as follows. When particles (i.e., quartz sand) collide with the coating, impact on the surface leads to the crater formation (Figure 5D, Stage 1). The extruded rim of the crater is susceptible to be removed by flowing particles (Stage 2), resulting in the formation of slender scratch/cutting marks (Stages 3–4). The continuous impact on the lips of the slender scratch causes random spreading to both sides (Stage 5), ultimately leading to the formation of a disorderly group of craters (Stage 6). In contrast, the 316L substrate exhibited a larger number of scratches and craters compared to the two coatings with a buffer layer (i.e., BMCP coating and TCE coating with a buffer layer), substantiating the effectiveness of the buffer structure in reducing the impact damage.

Figure 5E depicts that the erosion rate of the BMCP coating is 27.22 nm s^−1^, which is 71% lower than that of the 316L steel substrate, signifying an enhanced erosion resistance of the coating. During the repeated erosion in the testing loop, the microstructure displayed excellent resistance to vertical pressure and shear force, with the LA‐modified TiO_2_ NPs/CNTs between the microstructure framework remaining intact (Figure S13, Supporting Information). Furthermore, the erosion rate of the BMCP coating is reduced by 38.45% and 65.64% compared to the TCE coatings with a buffer layer and the BMC coating, respectively, further corroborating the superiority and importance of the microstructure and buffer layer.

## 3 Conclusion

In summary, a robust bionic microstructure integrated with a buffer layer was crafted, manifesting a high WCA of 154.4° and low water sliding angle of 3.8°. The introduction of conch‐shell‐inspired microstructures (i.e., cylinder‐shaped) greatly enhanced the wear resistance of the coating, effectively mitigating surface damage triggered by particle erosion. The optimal cylinder‐shaped microstructures with a diameter of 300 µm and an inter‐microstructure spacing of 300 µm, and the optimal the buffer layer with a pore size of 75 ± 15 µm, were identified via an integrated experimental and simulation study. Notably, the incorporation of LA‐capped TiO_2_ NPs, CNTs, and EP reinforced the mechanical properties of the coating. The resulting biomimetic microstructure coating with a porous design (BMCP) retained a superhydrophobicity even after experiencing sandpaper abrasion test, bending, and erosion loop test, manifesting exceptional mechanical durability. Additionally, it displayed remarkable resistance to corrosive media, including acidic, alkaline, and salt solutions, while maintaining excellent chemical stability in a diversity of extreme environments. Our design strategy, invoking the synergy of the metallic microstructures, the porous PDMS buffer layer, and the LA‐capped TiO_2_ NPs/CNTs/EP, for creating reliable superhydrophobic coating opens up an avenue to develop superhydrophobic materials with a multitude of highly desirable characteristics (e.g., mechanical durability, superhydrophobicity, deformability, chemical robustness, corrosion resistance, etc.). Such coatings hold great potential for a wide range of applications, including antifouling coating, drag reduction, and nondestructive transportation.

## Conflict of Interest

The authors declare no conflict of interest.

## Supporting information

Supporting Information

## Data Availability

The data that support the findings of this study are available in the supplementary material of this article.
