# Supplementary material for: A Robust Biomimetic Superhydrophobic Coating with Superior Mechanical Durability and Chemical Stability for Inner Pipeline Protection
Source: Adv Sci (Weinh). 2024 Jan 15;11(12):2305839. doi: 10.1002/advs.202305839 (PMC10966568; doi:10.1002/advs.202305839)
Supplement: Supplementary file 1 — Supporting Information [file ADVS-11-2305839-s001.pdf]

ADVANCED SCIENCE
Open Access

Supporting Information

for *Adv. Sci.*, DOI 10.1002/advs.202305839

A Robust Biomimetic Superhydrophobic Coating with Superior Mechanical Durability and Chemical Stability for Inner Pipeline Protection

Xuerui Zang, Jiang Bian, Yimeng Ni, Weiwei Zheng, Tianxue Zhu, Zhong Chen, Xuewen Cao, Jianying Huang*, Yuekun Lai* and Zhiqun Lin**

A Robust Biomimetic Superhydrophobic Coating with Superior Mechanical Durability and Chemical Stability for Inner Pipeline Protection

Xuerui Zang[a,b,c], *Jiang Bian*[a], *Yimeng Ni*[b], *Weiwei Zheng*[b], *Tianxue Zhu*[b], *Zhong Chen*[e], *Xuewen Cao*[a*], *Jianying Huang*[b,d*], *Yuekun Lai,*[b,d*], and *Zhiqun Lin*[c*]

[a] College of Pipeline and Civil Engineering, China University of Petroleum (East China), No. 66, West Changjiang Road, Huangdao District, Qingdao 266580, P. R. China

[b] College of Chemical Engineering, Fuzhou University, Fuzhou 350116, P. R. China

[c] College of Chemical and Biomolecular Engineering, National University of Singapore, Engineering Drive 4, Singapore 117585, Singapore

[d] Qingyuan Innovation Laboratory, Quanzhou 362801, P. R. China

[e] School of Materials Science and Engineering, Nanyang Technological University, 50 Nanyang Avenue, Singapore 639798, Singapore

Emails:

caoxw@upc.edu.cn (X. W. Cao); jyhuang@fzu.edu.cn (J. Y. Huang); yklai@fzu.edu.cn (Y. K. Lai); z.lin@nus.edu.sg (Z. Q. Lin)

Experimental Section

Materials

The microstructure of the 316L metal was prepared using an HBD-150 metal 3D printing instrument. Polydimethylsiloxane (PDMS) was sourced from Dow Corning, while multiwalled carbon nanotubes (CNTs), lauric acid (LA), and tetraethyl titanate were obtained from Aladdin. Epoxy resin (EP) was supplied by Zhenjiang Danbao Resin Co., Ltd., and polyurethane was acquired from Yichun Zhuoyue Chemical Co., Ltd. Anhydrous ethanol was obtained from Sinopharm Chemical Reagent Co., Ltd., and distilled water was produced using a UPHII-20T water purification system. Prior to use, the 316L metal was subjected to ultrasonication with acetone, ethanol, and deionized water for 15 min.

Optimization of surface metallic microstructure and buffer layer

The coating of superhydrophobic surfaces is required to display excellent wear resistance, resistance to environmental conditions, and enhanced durability. To attain this, in this work, the outer surface microstructure of the coating was formed via metal 3D printing (**Fig. 1,** illustration II), inspired from various biological skin surface shapes known for their exceptional wear resistance. The gap between the metallic microstructures was then filled with a mixture of LA-modified TiO_2 NPs and fibrous CNTs at a ratio of 2: 1 (**Fig. 1,** illustration III) together with EP (mixed with a polyurethane curing agent at a 1: 1 ratio of EP to curing agent). Notably, the combination of LA-modified TiO_2 NPs, CNTs, and EP resembles the reinforcement/fiber concrete structure, effectively enhancing the mechanical strength of the coating. The TiO_2 NPs were synthesized via a controlled hydrolysis-condensation reaction of tetraethyl titanate using

deionized water, absolute ethanol, and a small amount of hydrochloric acid for dilution and stirring. To ensure excellent dispersion, LA was dissolved in absolute ethanol to yield LA-capped TiO_2 NPs with the nonpolar groups of LA offering surface hydrophobicity.

To further enhance the mechanical properties of the coating, the structure of the intermediate layer was optimized. Citric acid monohydrate (CAM), known for its high solubility in water, was chosen as the pore-forming agent. PDMS (at a 30: 1 ratio to the curing agent ($C_6H_8O_7 \cdot H_2O$)) and CAM were thoroughly mixed at a 1: 2 ratio (**Table S1, Fig. 1,** illustration I). After curing at 100°C for 2 h, the mixture was ultrasonicated in an aqueous solution to yield a porous PDMS buffer layer.

Figures and Tables

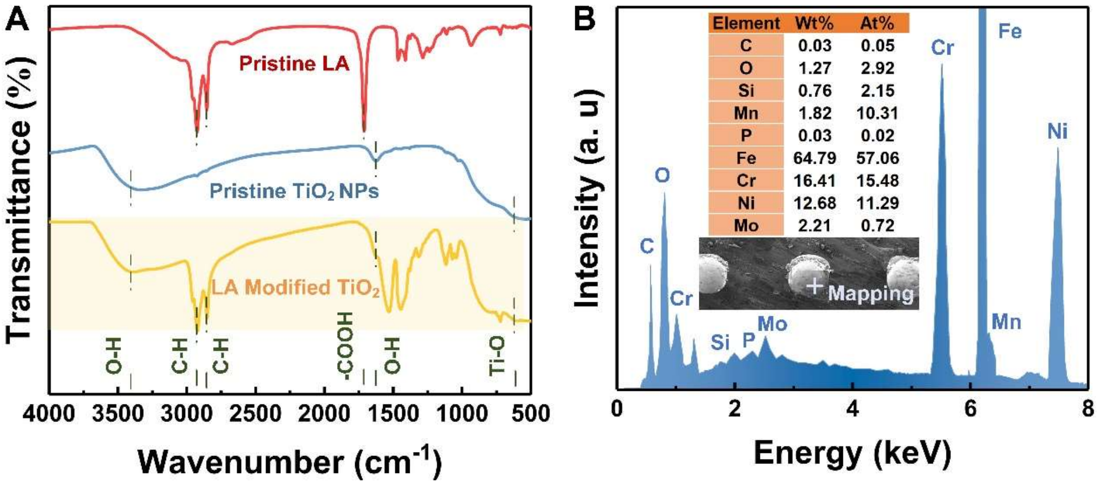

Fig. S1. (A) Fourier-transform infrared spectra (FTIR) of pristine and LA-capped TiO_2 NPs. **(B)** Energy dispersive X-ray spectroscopy (EDS) analysis of the BMCP on the surface of a cylinder-shaped microstructure (marked with a cross).

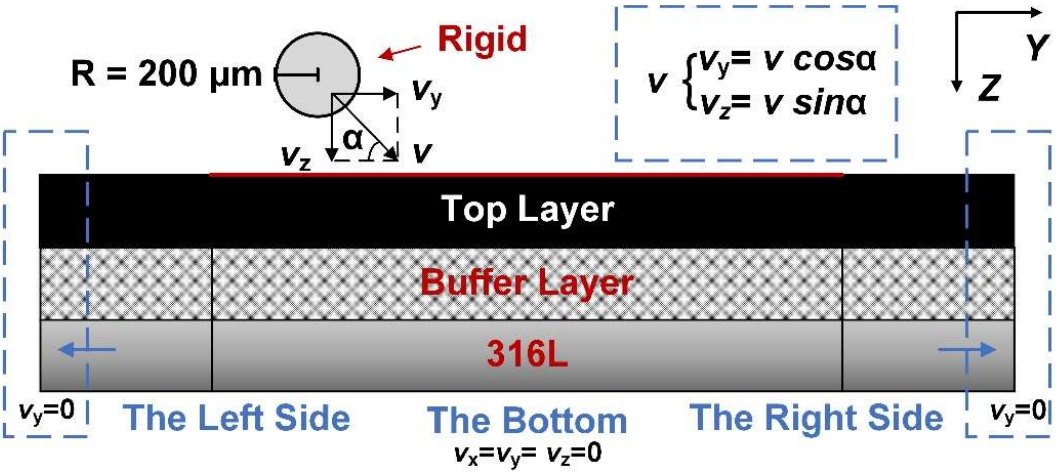

Fig. S2. The simulation details of the coating in ABAQUS software, where top layer, buffer layer and 316 L correspond to metal microstructures of varied sizes and shapes, PDMS buffer layer, and 316L metal, respectively.

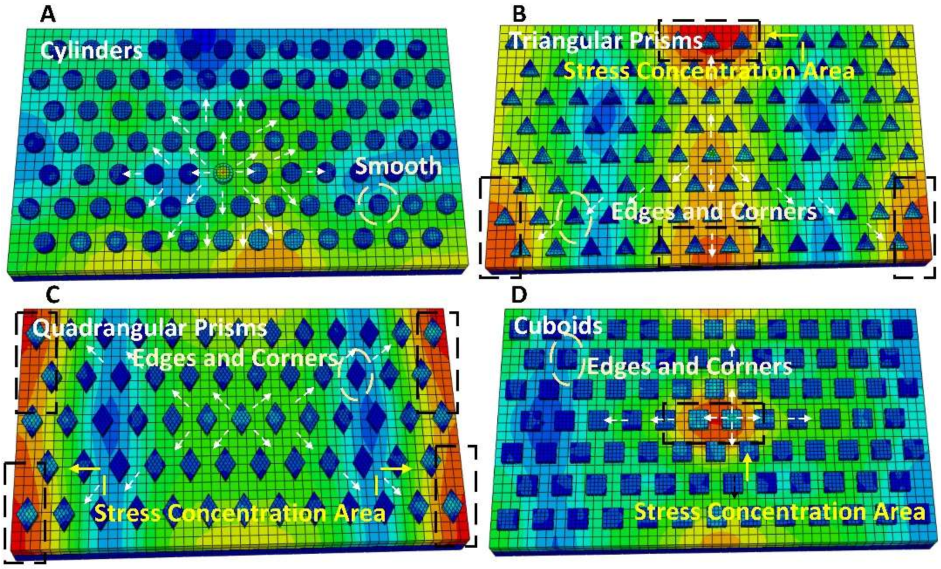

Fig. S3. Simulation results of microstructures with different shapes in a dislocation arrangement (i.e., hexagonal arrangement). **(A)** Cylinders. **(B)** Triangular prisms. **(C)** Quadrangular prisms. **(D)** Cuboids. Due to the different transmission directions of stress waves, the microstructure with edges and corners is more prone to stress concentration.

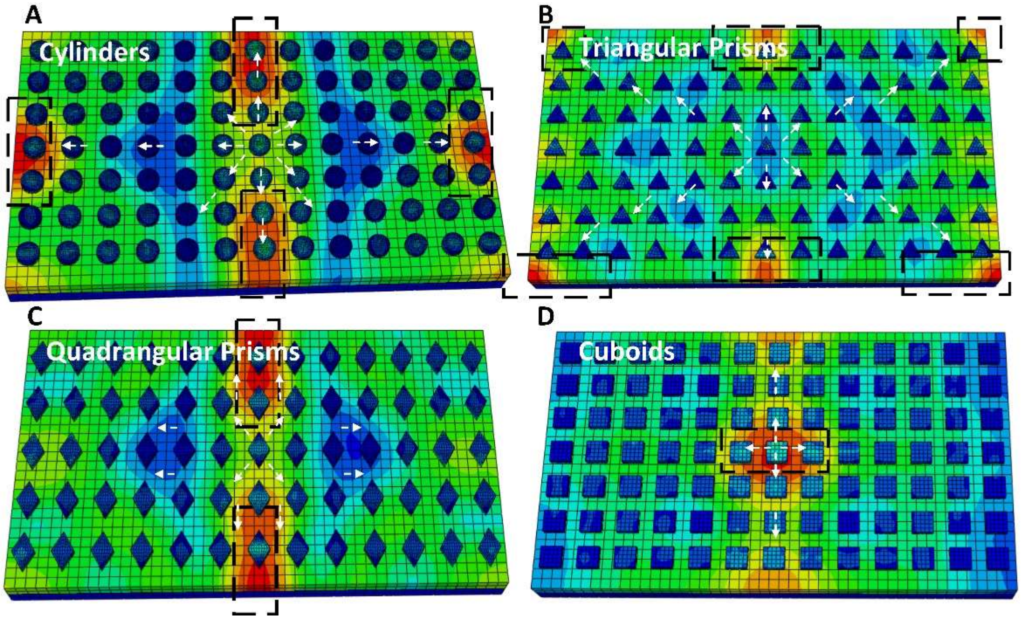

Fig. S4. Simulation results of with different shapes in a conventional arrangement (i.e., squared arrangement). **(A)** Cylinders. **(B)** Triangular prisms. **(C)** Quadrangular prisms. **(D)** Cuboids. The propagation ability of stress waves along the specified direction is enhanced by the squared arrangement, thereby increasing the stress concentration area on the material surface.

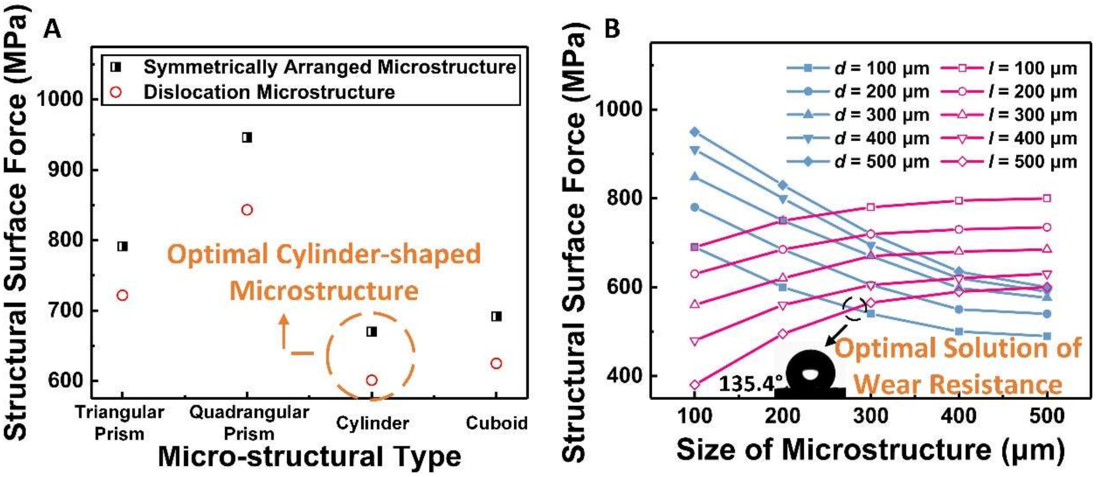

Fig. S5. Suitable surface microstructures identified via simulation. **(A)** Structural surface forces, depending on microstructures of different shapes. **(B)** The optimal cylinder-shaped microstructure (i.e., the smallest structural surface force, indicated by black dashed open circle), accessed via intersecting of the two curves (i.e., diameter-dependent and spacing-between-adjacent-cylinders-dependent structural surface forces), where a WCA of 135.4° (not superhydrophobic) was found.

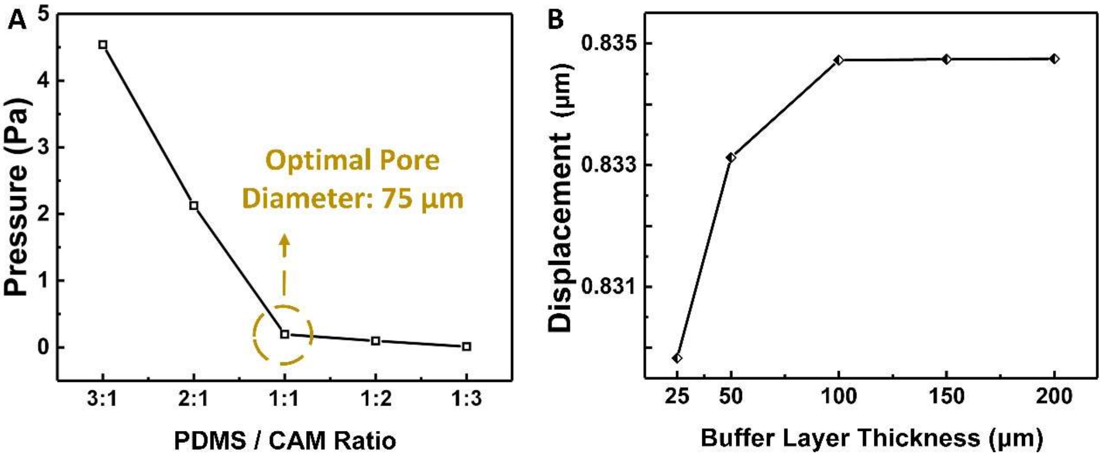

Fig. S6. Optimization of the PDMS buffer layer via simulation. **(A)** Mixing PDMS and CAM at varied mass ratios. **(B)** PDMS buffer layer thickness. The PDMS layer at the PDMS/CAM ratio of 1: 1 yields an optimal pore diameter of 75 μm. The PDMS layer at a thickness of 100 μm is found to reach the maximum displacement.

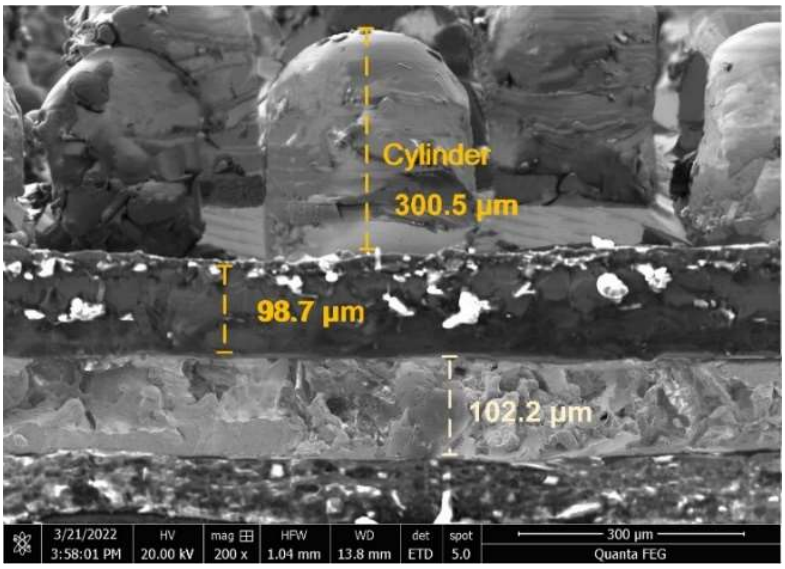

Fig. S7. Cross-sectional SEM image of a BMCP coating. The size of cylindrical metallic microstructure is 300.5 µm, a design guided by the simulation result (300 µm; **Fig. 2D**).

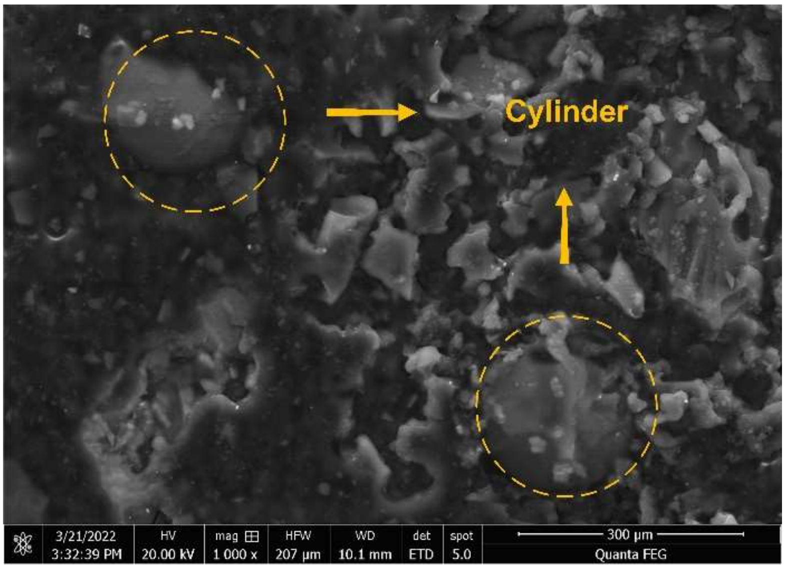

Fig. S8. Top-view SEM image of the BMCP coating surface, where cylinder-shaped microstructures are evident.

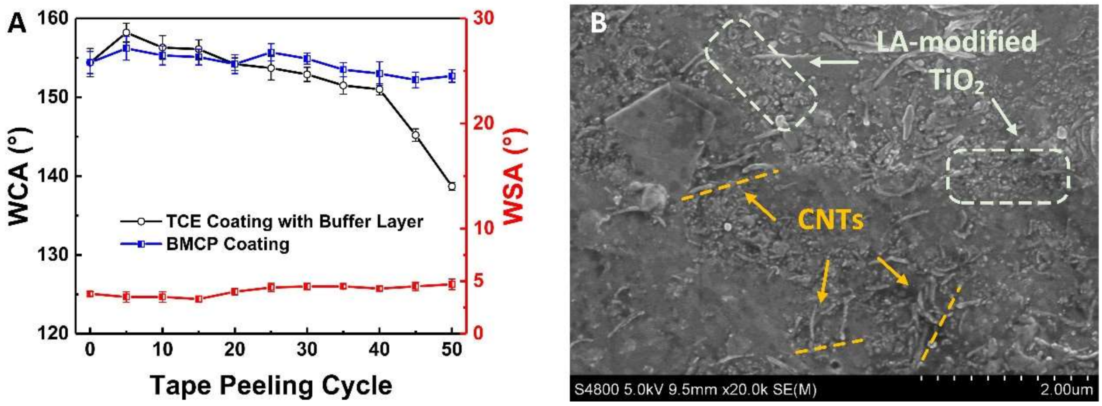

Fig. S9. Tape peel test. A tape peel test is used to examine the adhesion among the LA-modified TiO_2 NPs/CNTs, EP and microstructure. The test is performed by adhering a 3M tape to the coated surface, rolling back and forth over the tape surface with a 1 kg load, and then peeling the tape off the coated surface. **(A)** The relationship between the coating wettability and the number of the tape peeling cycle; **(B)** A SEM image of the coating surface after tape peeling, where the LA-modified TiO_2 NPs/CNTs is evident.

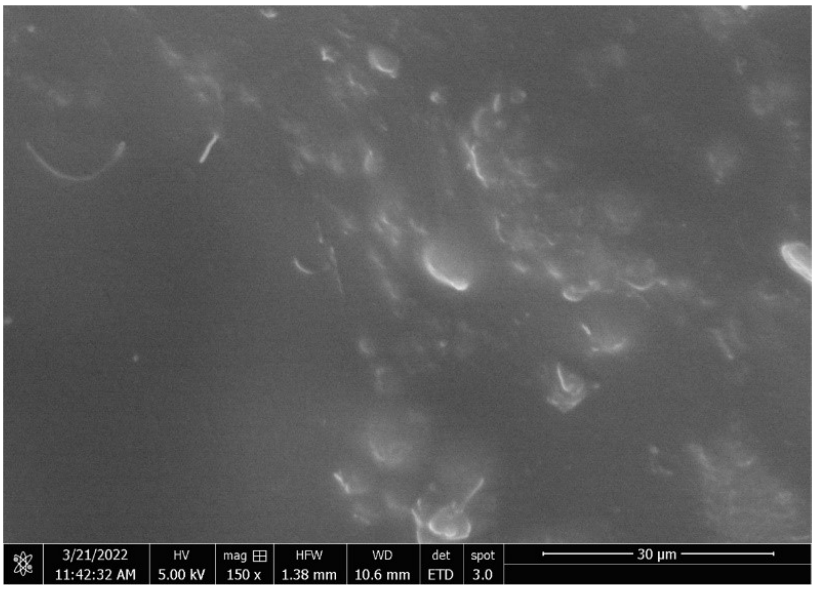

Fig. S10. The SEM image of the coating surface after 72 h immersion in pH=1 solution.

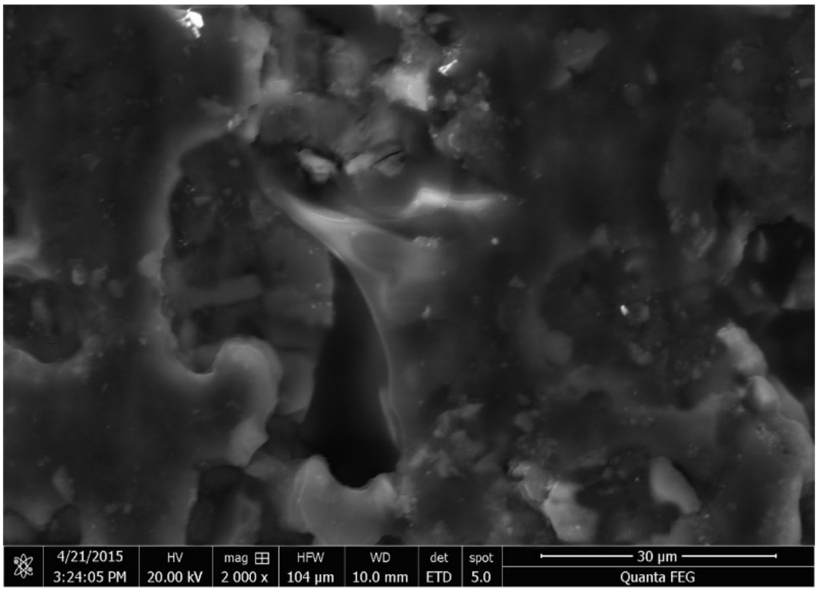

Fig. S11. The SEM image of the coating surface after 72 h immersion in pH=12 solution.

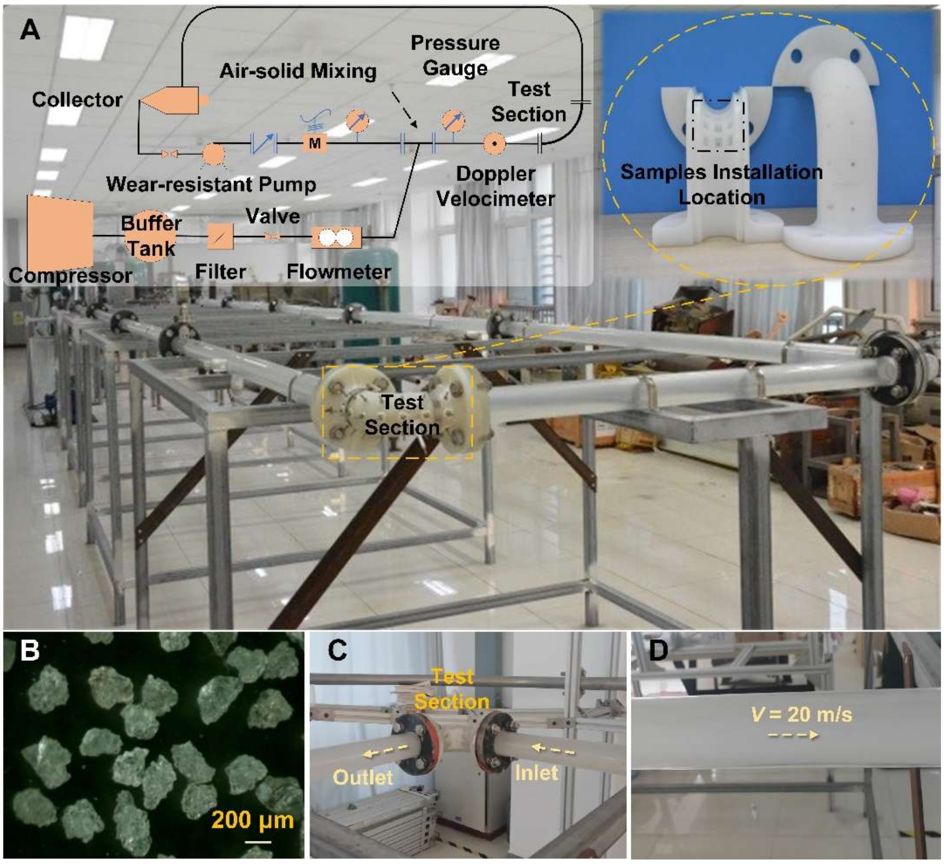

Fig. S12. Erosion test. (A) Erosion loop test. The test piece is fixed in the groove of the elbow to test the erosion resistance of the coating; **(B-D)** Quartz sand (B) for test and testing conditions (C-D).

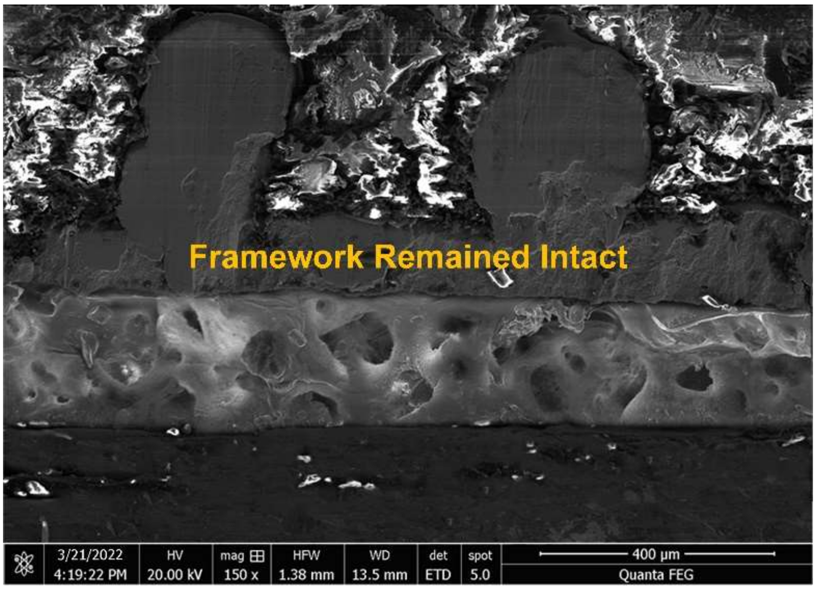

Fig. S13. The microstructure (upper part) of the coating after the erosion test.

Table. S1. Relationship between mass ratio and pore size

Mass Ratio (PDMS: CAM)	Pores (Size, μm)	PDMS (Mass, g)	CAM (Mass, g)	Buffer Layer (Length × Width × Height, mm)	Erosion Test (Maximum Erosion Rate after 12 h Cycle, nm/s)
3: 1	25 ± 5	0.5	0.17	2 × 2 × 2	41.37
2: 1	50 ± 10	0.5	0.25	2 × 2 × 2	34.59
1: 1	75 ± 15	0.5	0.5	2 × 2 × 2	27.22
1: 2	100 ± 25	0.5	1	2 × 2 × 2	28.63
1: 3	150 ± 25	0.5	1.5	2 × 2 × 2	27.11

※Maximum erosion rate occurs when the sample is positioned within the bend, and the sand particles impinge upon the sample at an angle of 22.5°. The erosion test findings reveal that by adjusting the PDMS: CAM ratio to 1:1, encompassing variations from 3:1 to 1:3 in a series of five experiments, the erosion rate of the coatings reached its lowest point at 27.22 nm/s. Notably, As the CAM contents increase, the erosion rate remains relatively constant, further affirming the optimal ratio between these constituents.

Table. S2. Influence of different preparation methods and mass ratios on the hydrophobicity and wear resistance of the coating

CNTs Content	Standing Water Contact Angle	Dynamic Water Contact Angle	Abrasion Resistance Test (600 Mesh Sandpaper, 2000 g (49 kPa))
0 g CNTs	159°	7.8°	600 wear cycles
20: 1 (TiO_2: CNTs)	157°	7.9°	900 wear cycles
4: 1 (TiO_2: CNTs)	153°	8.2°	1100 wear cycles
2: 1 (TiO_2: CNTs)	154°	8.7°	1200 wear cycles
1: 1 (TiO_2: CNTs)	134°	20.8°	700 wear cycles

Table. S3. The relationship between pore size and impact resistance

Mass Ratio (PDMS: CAM)	Erosion Duration (h) 12		24		36		48	
	WCA (º)	WSA (º)	WCA (º)	WSA (º)	WCA (º)	WSA (º)	WCA (º)	WSA (º)
Pure PDMS	155.2	6.7	154.1	7.5	148.7	9.3	145.2	12.8
3: 1	156.2	6.7	153.6	6.6	150.8	7.4	147.3	10.0
2: 1	154.2	6.7	154.4	5.9	152.5	6.8	149.4	11.5
1: 1	155.7	6.7	154.9	7.2	152.3	7.5	151.7	8.3
1: 2	154.7	6.7	155.3	7.7	152.8	7.4	150.8	9.2
1: 3	155.1	6.7	153.6	6.1	150.2	7.1	151.2	8.7
